# Notes on Typhoid Fever: Tropical Life and Its Sequelœ

**Published:** 1891-06

**Authors:** 


*Notes on Typhoid Fever: Tropical Life and its Sequelæ.* By JEFFERY A. MARSTON, M.D., C.B. London: H. K. LEWIS. 1890.

These *Notes*, written from the military medical officer's standpoint, are a few "dropped stitches in the pathological pattern of typhoid fever." Two points bearing on treatment are worthy of comment: the one is the omission of all mention of intestinal antiseptics with a view to the prevention of relapses, and the other is the great utility of systematic antipyretic treatment either by drugs or (if these fail) by means of the cold bath.

The two chapters on tropical life will be of interest to those who have not seen much of life and disease in the tropics.

